# Social networking in mental health interventions for adolescents

**DOI:** 10.1177/1757913920924431

**Published:** 2020-10-01

**Authors:** E Sanger

**Affiliations:** University of York, York YO10 5DD, UK

This article looks at the benefits of using social media in providing access to mental health support for adolescents, due to the anonymity and improved sense of community that social networking allows.

Adolescents are susceptible to mental health problems due to ‘multiple physical, emotional and social changes’^1^ as they adapt to new responsibilities and relationships. Globally, 10%–20%^2^ of adolescents suffer with their mental health which can impact on relationships, school and physical health.^3^ All of this can continue to impact people in adulthood, with 75% of mental illness in adults coming on before 18 years.^4^ There are many factors that can contribute to development of mental health problems, including peer-pressure, exploring sexual identity and relationships with friends and family.^1^

There is a lot of debate about the impact social media can have on the mental health of adolescents. While some papers have shown links to social media use and depressive symptoms,^5,6^ social media can be used to benefit the mental health of adolescents. Social media can be used to strengthen relationships with both new and existing friends online, reducing feelings of isolation and loneliness.^7^ Being online allows people to express their feelings more easily and receive support from others.^7,8^ The benefits of this are clear, as a review found access to more support online reduced the levels of depression and anxiety.^9^

Finding more ways to provide help with mental health is crucial, as services are limited, and young people are struggling with accessing help.^10^ There are more than two billion people active on social media.^11^ Clearly, this presents a chance for social media platforms to reach a large number of people with information on mental health. In fact, 72% of adults using the Internet in the US have searched health issues online.^11^ In particular, mobile technology is becoming more popular with 96% of people aged 12–17 years using a mobile^12^ and more apps are becoming available for disseminating information on health care.^3^


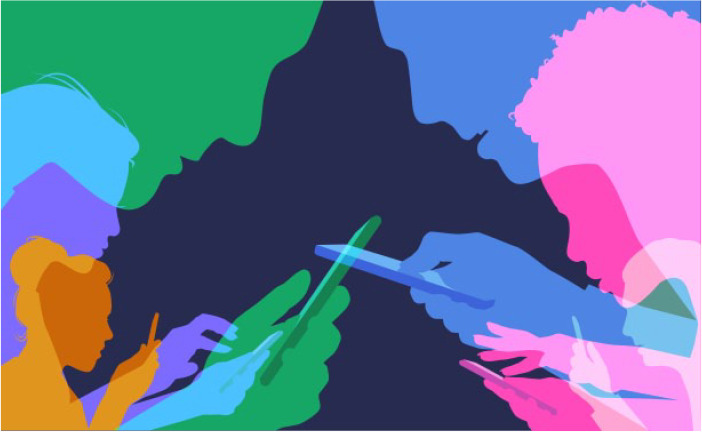


Therefore, social media platforms can be used as part of a mental health service by providing more education and awareness of mental health.^13^ It has been shown that young people find interventions online engaging and highly usable.^13^ Social media is unique in that it provides an opportunity to reach a breadth of people with ease. There is a larger capacity to provide treatment through digital mental health care, as you do not face the ‘geographical barriers’^3^ that you do with face-to-face treatment. Furthermore, being online allows people to remain more anonymous,^13^with privacy being an important factor to adolescents using discussing health online.^3^ This allows young people to talk about their mental health online without fear of being judged, helping overcome the stigma of mental illness, a large barrier in seeking help.^3^ They can also communicate with people with similar conditions, developing supportive networks,^7^ which can help reduce feelings of loneliness^9^. Therefore, social media may be useful for targeting people who would not usually seek help.^3^ A review found that use of social media in mental health was associated with high engagement rates and low dropout, with the most highly rated social media interventions involving a moderator in the group.^13^ Thus, mental health interventions involving social networking can successfully exploit the increasing use of social media sites to impact mental health in adolescents in a positive way. It improves access to interventions, allows anonymity and creates a stronger sense of community to discuss mental health.
